# Correction: KSR1- and ERK-dependent translational regulation of the epithelial-to-mesenchymal transition

**DOI:** 10.7554/eLife.99343

**Published:** 2024-05-07

**Authors:** Chaitra Rao, Danielle E Frodyma, Siddesh Southekal, Robert A Svoboda, Adrian R Black, Chittibabu Guda, Tomohiro Mizutani, Hans Clevers, Keith R Johnson, Kurt W Fisher, Robert E Lewis

**Keywords:** Human

Rao C, Frodyma DE, Southekal S, Svoboda RA, Black AR, Guda C, Mizutani T, Clevers H, Johnson KR, Fisher KW, Lewis RE. 2021. KSR1- and ERK-dependent translational regulation of the epithelial-to-mesenchymal transition. *eLife* **10**:e66608. doi: 10.7554/eLife.66608.

Published 10 May 2021

We were alerted by a PubPeer thread about a partial overlap of two representative images that reveals a duplication affecting two fields in Figure 5A (Lamin β2, HCT116 and SW480) and Figure 5—figure supplement 1C (microscope images N-cad OE: siControl/siEPSTI1).

For Figure 5A, at the error originated from improper cropping of the loading control lamin β2 blot image intended to create a lamin β2 image for the middle panel (SW480). We identified the samples loaded in the respective lanes associated with SW480 and used it generate the corrected images in Figure 5A. We also acknowledge the uneven loading lamin β2 in the samples marked with SW480 Con and KSR1 CRISPR. We quantified the relative density of EPSTI1 with Lamin β2 using the corrected data. The quantification results are provided below. This correction does not alter the conclusions stated in the results.

**Figure 1:**
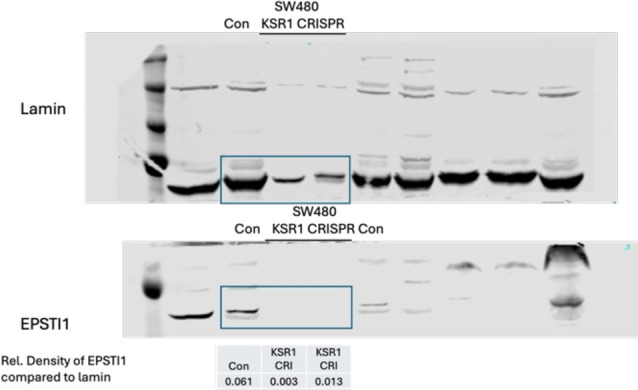


For Figure 5—figure supplement 1C, we suspect that the duplication occurred unintentionally due to errors during the preparation of the figures. The respective correct images have been identified and used to generate corrected images in Figure 5—figure supplement 1C. This error does not impact the quantifications associated with Figure 5—figure supplement 1C, nor does it affect the conclusions of the manuscript. The inclusion of identical lanes/microscopes images in these figures was an unintentional oversight.

The corrected Figure 5 is shown here:

**Figure 2:**
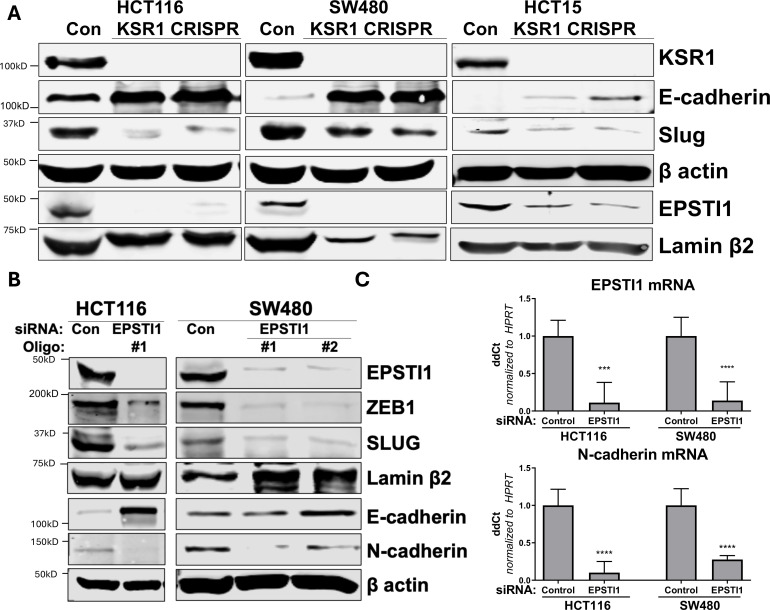


The originally published Figure 5 is shown for reference:

**Figure 3:**
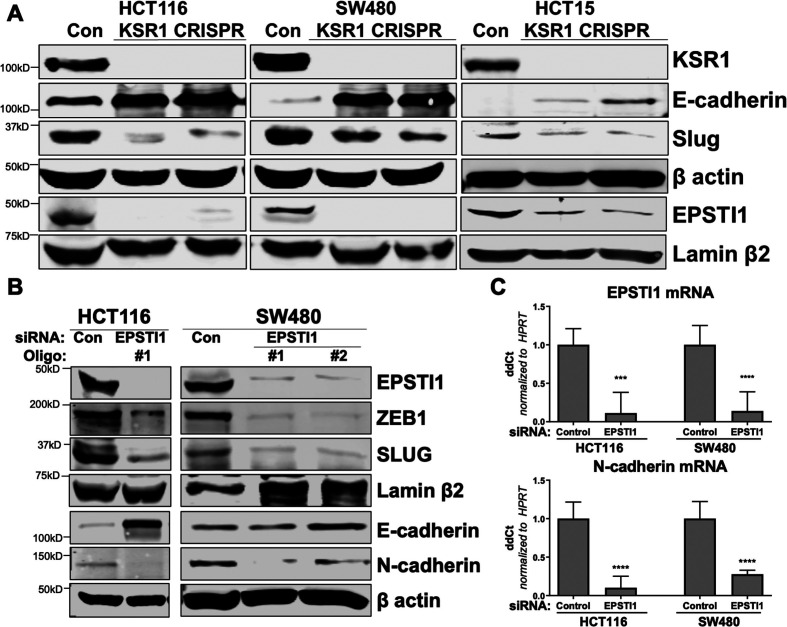


The corrected Figure 5—figure supplement 1 is shown here:

**Figure 4:**
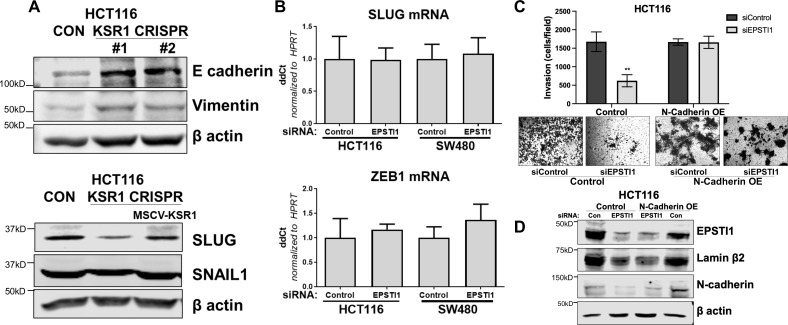


The originally published Figure 5—figure supplement 1 is shown for reference:

**Figure 5:**
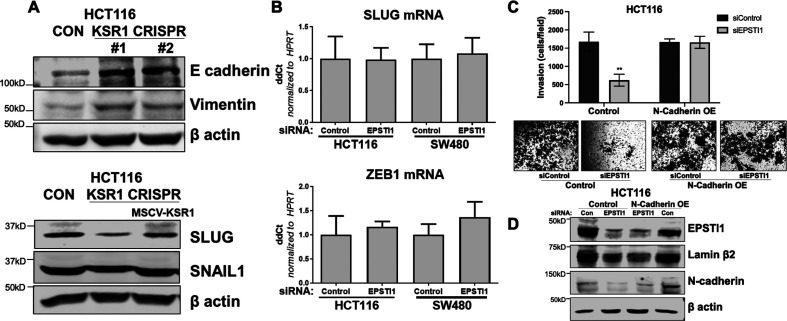


The corrected Key Resources Table entries with revised sequences are shown here:

**Table:** 

Reagent type (species) or resource	Designation	Source or reference	Identifiers	Additional information
Transfected construct (*Homo sapiens*)	KSR1 sgRNA#1	This paper	pCAG-SpCas9-GFP-U6-gCR1.1	TTGGATGCGCGGCGGGAAAG
Transfected construct (*Homo sapiens*)	KSR1 sgRNA#2	This paper	pCAG-SpCas9-GFP-U6-gCR1.2	CTGACACGGAGATGGAGCGT

The originally published Key Resources Table entries are shown for reference:

**Table:** 

Reagent type (species) or resource	Designation	Source or reference	Identifiers	Additional information
Transfected construct (*Homo sapiens*)	KSR1 sgRNA#1	This paper	pCAG-SpCas9-GFP-U6-gCR1.1	GTGCCAGAAGAGCATGATTTT
Transfected construct (*Homo sapiens*)	KSR1 sgRNA#2	This paper	pCAG-SpCas9-GFP-U6-gCR1.2	GTGCCAGAAGAGCATGATTTT

We thank the users of PubPeer for alerting us of this issue. The article has been corrected accordingly.

